# Can Africa achieve herd immunity?

**DOI:** 10.1186/s41256-021-00231-1

**Published:** 2021-12-02

**Authors:** Don Eliseo Lucero-Prisno, Isaac Olushola Ogunkola, Ekpereonne Babatunde Esu, Yusuff Adebayo Adebisi, Xu Lin, Hao Li

**Affiliations:** 1 Department of Global Health and Development, London School of Hygiene and Tropical Medicine, London, UK; 2 Faculty of Management and Development Studies, University of the Philippines Open University, Los Baños, Laguna Philippines; 3 Department of Public Health, University of Calabar, Calabar, Nigeria; 4 Faculty of Pharmacy, University of Ibadan, Ibadan, Nigeria; 5 Department of Thoracic Surgery, The First Affiliated Hospital, School of Medicine, Zhejiang University, Hangzhou, Zhejiang China; 6 Global Health Institute, Wuhan University, Wuhan, Hubei China

**Keywords:** Africa, COVID-19, Herd immunity, Vaccine

## Abstract

The World Health Organization described herd immunity, also known as population immunity, as the indirect fortification from an infectious disease that happens when a population is immune either through vaccination or immunity developed through previous exposure to infection. The emergence of COVID-19 vaccine is a step towards the achievement of herd immunity. Over one billion people across the globe have been vaccinated and Africa recorded only 2%. The objective of this article was to develop a forecast of the number of people to be vaccinated to achieve herd immunity in the 13 WHO-identified priority African countries for COVID-19. Herd immunity is achieved when one infected person in a population causes less than one secondary case on average, corresponding to the effective basic reproduction number (R_0_). Vaccine delivery and distribution infrastructure including the cold chain remains weak. Vaccine hesitancy is also one of the limiting factors that may hinder herd immunity in Africa. In order to achieve herd immunity globally, African countries should not be excluded in fair and equal distribution of vaccines. Relevant stakeholders should foster commitment as well as community sensitization on COVID-19 vaccines and integration of COVID-19 vaccines in existing healthcare services.

## Introduction

As of 2021 July, the notorious virus COVID-19 has spread globally at a daunting speed, causing millions of cases and more than three million deaths in just a year and few months [1]. The world was thrown into abject confusion as the virus could not be contained in almost all countries of the world, including those with world-class health facilities [2]. The pandemic did not just affect the health sectors of countries alone but also the social and economic aspects. The pandemic was highly felt across different socio-economic strata, particularly those belonging to vulnerable groups such as the poor, like street vendors and other marginalized groups [2, 3]. The scientific world experienced a breakthrough when diverse COVID-19 vaccines were developed within a year—an unnatural speed for vaccine development. This exemplifies the tenet that the role of vaccines in mitigating disease morbidity and mortality around the globe cannot be overemphasized. There are current debates on how Africa will possibly acquire enough vaccines to cater for its population of 1.3 billion [4, 5].

Fast forward to 2021, the World Health Organization (WHO) partnered with relevant stakeholders to distribute vaccines to all countries, including Low-and-Middle Income Countries (LMICs). The WHO approved the first vaccine for emergency use, the world reached a milestone of vaccinating one billion people across the globe within four months [6]. Before the distribution of the vaccines, global health and public health experts launched a herd immunity defence strategy that aroused interest and controversy in the international community. The concept of herd immunity has been of great importance, mostly in animal husbandry.

According to WHO, herd immunity also referred to as population immunity, is the indirect fortification from an infectious disease that happens when a population is immune either through vaccination or immunity developed through previous exposure to infection [7]. Vaccines train our immune systems to create antibodies that fight disease, just as would happen when exposed to a disease, but—vitally—vaccines work without making us sick. Vaccinated people are protected from getting the disease in question and passing on the pathogen, breaking any infectious chains of transmission. The big question is can Africa achieve herd immunity even as COVID-19 vaccines are being rolled out?

With the present inequality in vaccine distribution, there are possibilities that the virus may not be curbed easily in most African countries. More than three-quarters of all doses of vaccines being used so far have been administered in just ten countries of the world. People in more than 170 nations are to struggle for the remainder [6]. The irregular distribution of vaccinations, both in and between nations, threatens to slow progress towards the achievement of herd immunity. The United States and China alone account for closely half of all the doses given out.

On the other hand, just 2% have gone to the entire continent of Africa [6]. In the early days of the pandemic, the WHO identified 13 priority African countries for COVID-19, which included Algeria, Ghana, South Africa, Tanzania, Kenya, Mauritius, Angola, Cote d’Ivoire, Ethiopia, the Democratic Republic of the Congo, Nigeria, Zambia, and Uganda [8]. The objective of this article was to develop a forecast of the number of people to be vaccinated to achieve herd immunity in the 13 WHO-identified priority African countries for COVID-19.

## COVID-19 basic reproduction number and the forecast

Herd immunity is achieved when one infected person in a population causes less than one secondary case on average, corresponding to the effective basic reproduction number (R_e_) [9–11]. R_e_ may contrast across populations and over time, depending on the characteristics and number of contacts among individuals and hypothetically environmental factors [10]. Arithmetically, the herd immunity threshold is defined by 1 − (1/R_o_) [9]. For example, R_o_ =3, the corresponding herd immunity threshold is 0.67, and that implies that 67% of the population must have been protected either through vaccination or previous exposure to achieve herd immunity [9, 10]. COVID-19 has been calculated from the start to have a basic reproduction number (R_o_) between 2 and 6 [9–11], with differing values across countries.

The differences in the basic reproduction value is consequently a reflection of the deviation of the transmission dynamics of the COVID-19 infection across countries. If the acquired immunity of a community rises above 67%, then a gradual decline in the number of incident cases is recorded. With direct impacts on the R_o_ and herd immunity threshold, the spread of any infectious disease, as in this case, COVID-19, depends on factors such as the population structure, population density, and differences in the contact rates across existing demographic groups [9, 10]. This applies to both immunity via vaccination and previous exposure to infection. Although, it is a fact that herd immunity through exposure may lead to a high mortality rate, especially among high-risk groups. Achieving herd immunity should be achieved by protecting people through vaccination, not by exposing them to the pathogen that causes the disease.

The percentage of people who need to be immune to achieve herd immunity varies with different diseases. For example, herd immunity for polio is about 80%. Herd immunity against measles requires about 95% of a population to be vaccinated. The remaining 5% will be protected because measles will not spread among those who are vaccinated. The proportion of the population that must be vaccinated against COVID-19 to begin inducing herd immunity is not known. However, there have been numerous epidemiological studies geared to understand the transmission dynamics of COVID-19 [11]. This can be quantified by the basic reproduction number and the serial interval, which is the time delay between the symptom onset of a primary case and his/her secondary case [10, 11]. A high reproductive value and short serial interval imply rapid growth because the secondary infections generated from an initial case at the beginning of an epidemic keeps spreading with a short time. An estimated exponential growth rate and the basic reproduction number of the COVID-19 at the early stage of the pandemic in Africa was reported to be 0.22 per day and 2.37, respectively [11]. In this study, the estimated basic growth rate of 2.37 will be used to make our forecast. Some global health experts have opined that herd immunity can only be achieved by 90% vaccination of the world’s population [6, 7, 9, 11]. This coverage will give a basic reproductive number (R_o_) of 10.

In this study, the aggregate number of confirmed cases of COVID-19 and deaths were collected from publicly available data of the outbreak situation report of the WHO Coronavirus Disease (COVID-19) Dashboard on the 3rd of May 2021 [4, 12]. The country’s population was obtained from the website of the Population Reference Bureau [4]. We forecasted the number of people to be vaccinated for herd immunity to occur using R_0_ to be 2.37 and 10 giving a projection of 57.8% and 90% coverage, respectively. The case fatality rate was calculated using the total number of deaths and cumulative cases reported in each region on the 3 May 2021.

Table 1 shows the population distribution in the WHO identified 13 priority African countries for COVID-19, cases, death, case fatality rate of COVID-19 and the total number of vaccine doses administered as of 28 of April 2021. The country with the highest number of cases of COVID-19 is South Africa. South Africa has a population of 59.6 million with a total number of 1,582,842 confirmed cases of COVID-19 and over 54,000 deaths with a case fertility rate (CRF) of 3.4%. Though 182,983 doses of vaccines have been administered in South Africa. Albeit, our forecast estimates that the country will need to vaccinate 34,448,800 people at R_o_ = 2.37 or 53, 640, 00 people at R_o_ = 10 to achieve herd immunity. Tanzania has the lowest cases of COVID-19 with a total of 509. The country has a population of 59.7 million and has recorded 21 deaths as of the 3rd of May with a CFR of 4.1%. There is no known fact concerning vaccination in the country as at the time of writing this paper. Notwithstanding, our forecast estimates the country at R_o_ 2.37, 10 will need to vaccinate a total of 34,506,600 and 53,730,000 respectively to achieve herd immunity. Africa would need 1.6 billion doses if it uses a double-dose regime, or 800 million doses for a single-dose regime so as to vaccinate 60% of the African continent.

**Table 1:** Population distribution of the WHO identified 13 priority African countries for COVID-19, total cases, deaths, CFR, number of vaccine doses administered and projected number of people to be vaccinated

Countries	Population as at mid-2020 in million	Total number of reported cases	Deaths	Case Fatality rate	Total number of vaccine doses administered as of 28 April 2021	Projected number of vaccinated population to achieve herd immunity (*R*_0 =_ 2.37) 57.8%	Projected number of vaccinated population to achieve herd immunity (*R*_0 =_ 10) 90%
Algeria	44.4	122,311	3261	2.7	Pending	25,663,200	39,960,000
Angola	32.5	26,815	600	2.2	443,263	18,785,000	29,250,000
The Democratic Republic of Congo	89.6	29,904	768	2.6	1075	51,788,800	80,640,000
Cote D’Ivoire	26.2	46,114	286	0.6	118,224	15,143,600	23,580,000
Ethiopia	114.9	258,062	3709	1.4	1,029,184	66,412,200	103,410,000
Ghana	31.1	92, 601	779	0.8	842,521	17,975,800	27,990,000
Nigeria	206.1	165,153	2063	1.3	1,188,839	119,125,800	185,490,000
Mauritius	1.3	1206	16	1.3	197,646	751,400	1,170,000
South Africa	59.6	1,582,842	54,406	3.4	182,983	34,448,800	53,640,000
Tanzania	59.7	509	21	4.1	–	34,506,600	53,730,000
Kenya	53.5	160,053	2744	1.7	827,671	30,923,000	48,150,000
Uganda	45.7	41,907	343	0.8	232,631	26,414,600	41,130,000
Zambia	18.4	91,670	1251	1.4	17,851	10,635,200	16,560,000
Total	783	2,619,147	70,247	2.7	5,081,888	452,574,000	704,700,000

The overall CFR of COVID-19 is 2.7% in the 13 priority African countries for COVID-19. However, 5,081,888 doses of vaccines have been administered in almost all of these countries as of 28 April 2021. It is not enough to drive herd immunity in the region. At a R_o_ = 2.37 and 10, a total of 452,574,000 and 704,700,000 people respectively need to be vaccinated in those prioritized region to drive herd immunity. If herd immunity can be achieved, the present inequality in vaccine distribution may disrupt the progress. This will affect both the African countries and high-income countries as unequal access to vaccines in any part of the world may drive variants of COVID-19.

## Vaccine hesitancy: a threat to herd immunity in Africa

WHO listed the Sinopharm COVID-19 vaccine for emergency use on the 7th of May, 2021 in addition to Oxford–AstraZeneca, Pfizer-BioNTech, Moderna, Sinovac, and Johnson & Johnson. Though countries in Africa still have short supply of vaccines. Aside inadequate vaccines in Africa some factors such as vaccine hesitancy may disproportionately hinder the progress towards herd immunity too. Vaccine hesitancy is defined as the delay in the acceptance, or blunt refusal of vaccines and has been identified as a growing trend in global health [13]. Even though the continent has achieved impeccable progress since the Expanded Programme on Immunization (EPI) launch, hesitancy is threatening the progress made in the past years [13, 14]. There have been various polemics on vaccines in Africa [13]. This has led people to delay or refuse the uptake of endorsed vaccines. This refusal usually has a ripple effect when it is coming from an adult or an influencer. For example, An African mother or father who rejects vaccination will never allow his or her children to be given the vaccine if these individuals are in a place of authority in their community. This refusal may transcend to a majority of the members of that community.

Vaccine hesitancy poses significant threats not only for the hesitant individual but also to the broader community [13, 14]. Delays and refusals of vaccination make communities unable to reach thresholds of vaccine uptake that confer herd immunity, hence raising the possibility of an outbreak should a vaccine-preventable organism begin spreading in that community. One of the fueling cause of vaccine hesitancy in Africa is misinformation. Since the advents of COVID-19 in Africa, there have been false rumours and conspiracy theories against the use of vaccines [13]. The use of any COVID-19 vaccines has been associated with various rumours ranging from DNA alteration to inserting microchip to monitor Africans and depopulate the continent. Misinformation has a strong connection with hesitancy, and it is a massive hindrance to the achievement of herd immunity. The depletion of vaccines available for developing countries, especially for African countries, may also affect herd immunity in the continent. Some countries are hoarding vaccines, with rich nations such as Switzerland amassing more than seven times the amount needed to immunize their entire population if all the vaccines are approved [15]. This act may reduce the number of vaccines available to African countries. The hoarding of vaccines may pose a serious challenge than its discovery.

## Vaccine equity in rural Africa

It is no longer news that most people in Africa inhabit rural settings. These rural communities have limited basic amenities [16]. The challenge of water supply, electricity, roads, schools, and health centres have always been pointed out as significant limitations affecting rural communities. Getting vaccines down to rural communities in Africa might be faced with some inherent challenges, one of which is difficult terrain [16]. Rural communities in Africa usually lack good roads, and it may be challenging for health workers to access these communities. In Africa, hard-to-reach areas will continue to exist, and their underserved populations are vulnerable to infectious diseases [16]. Getting vaccines across to the hard-to-reach communities takes time due to poor or nonexistent roads, difficult terrain, and long distances to travel. The inequities in accessing hard-to-reach areas have grave implications for the control and prevention of vaccine-preventable diseases, especially in the case of coronavirus disease [16]. Poor information access, infodemic and poor health literacy in rural areas of Africa have been a challenge [16]. These challenges will hinder the achievement of herd immunity in Africa if proper structure and strategies are not employed to tackle them.

Achieving vaccine equity in Africa may also be hampered by a lack of supply cold chain. Delivering vaccines to all corners of Africa is a complex undertaking. It takes a chain of accurately harmonized events in temperature-controlled environments to store, manage and transport these essential products. The power supply and shortage issue in Africa will hugely affect supply cold chain, especially in hard-to-reach areas that lack basic amenities [16]. The cost of acquiring vaccines, setting up good logistic chain supply and delivery may be too exorbitant for most African countries to afford [5]. The phenomenon of herd immunity is only achievable if total equity is involved in vaccine distribution.

## A battle of speed and mutation

Viruses constantly change and become more diverse, and COVID-19 is not an exception. In fact, in the current global crisis, SARS-CoV-2 has been mutating at a rate of about 1–2 mutations per month [17–19]. Several variants have lately made headlines, including the deadly Delta variant, a South African variant known as 501Y.V2 or B.1.351 lineage, a UK variant known as 501Y.V1, VOC 202,012/01, or B.1.1.7 lineage, a Brazilian variant known as 501Y.V3 or P.1 lineage, California variants known as B.1.427 and B.1.429, and many others [18, 19]. These variants seem to spread more easily and quickly than other variants, which may lead to more cases of COVID-19. An increase in the number of cases will put more strain on healthcare resources, lead to more hospitalizations, and potentially more deaths especially in Africa with already at-capacity healthcare systems.

Geneticists has said that, two SARS-CoV-2 collected from anywhere in the world differ by an average of just 10 RNA letters out of 29,903 [18]. This may have no serious consequences, that is, the virus ability to spread or cause death if they do not alter the shape of a protein, whereas those mutations that do change proteins are more likely to inhibit a possible achievement of herd immunity [18]. The effect of COVID-19 mutation may usher a catastrophe that may overpower the continent if care is not taken. Some of the potential consequences that makes COVID-19 mutation a public health concern include;

- The ability of possible new variants to spread more quickly with high speed among the people.
- The ability to cause more serious diseases within the population leading to higher mortality.
- The ability to dodge detection by specific viral diagnostic tests which may reduce testing rate.
- Declined susceptibility to therapeutic agents such as monoclonal antibodies.
- The ability to dodge natural or vaccine-induced immunity [17, 18].

Among all of the aforementioned potential consequences, the ability of variants to dodge vaccine-induced immunity—would likely be the most disconcerting. When a substantial percentage of the population is vaccinated, there are possibilities of immune pressure that could indulge and accelerate emergence of such variants [18]. This could become worrisome mutations, because the antibody generated from the administration of vaccine could be thwarted by a single viral mutation. A serious mutation may hinder the achievement of herd immunity. A fast and well-paced vaccination programme in Africa may bypass the strain of COVID-19 and ultimately lead to herd immunity in the long run.

## Available help in Africa

The Africa continent is home to over one billion people as of mid-2020 with a projection of hitting over two billion by mid-2050 [4]. One may doubt such increment in population with the present realities of COVID-19 pandemic. Though the continent recorded a lesser mortality rate compared to western countries. At first, there were speculations from global health experts on how the continent will be worst affected by the pandemic, but counterintuitively, the pandemic was handled with minimal casualties [3]. Prior to the discovery of safe and effective COVID-19 vaccines, most African countries signed up to a ground-breaking initiative, which aims to secure at least 220 million doses of the vaccine for the continent, once licensed and approved [5]. In fact, one can conclude that the COVAX initiative aside the support of China and other HICs was one of the brilliant initiatives in terms of vaccine equity and distribution in the continent.

COVAX is a ground-breaking global initiative which include African countries and ensure they are not left at the back of the queue for COVID-19 vaccines. As the global rollout of COVAX vaccines accelerates, the first COVID-19 vaccination campaigns in Africa using COVAX doses began on 1 March 2021 in Ghana and Côte d’Ivoire [12]. Through this initiative, a few millions of vaccines have been distributed to many African countries. The initiative also ensured that vaccines that have passed regulatory approval or WHO prequalification are delivered equally to all participating countries, proportional to their populations [12]. The initial quota prepared for Africa by COVAX is to give 20% of the total population vaccines. The recent Paris Summit proposed an increment from 20 to 40%. Though no concrete commitment were made at the Summit.

As suggested by Lucero-Prisno et al., capable countries should help in providing vaccines to LMICs to accelerate equitable access to vaccines globally [5]. The government of Sweden made a donation of a million doses of COVID-19 vaccines through the COVAX initiative to aid African countries and other LMICs [20]. There are speculations of vaccine commitment coming from the G7 (Canada, France, Germany, Italy, Japan, the United Kingdom and the United States) [21]. A commitment to give one billion doses of COVID-19 to poor countries in which Africa will benefit from [21], but the challenge is when will they become available. China not only donated vaccines to more than 40 African countries and the Africa Union Commission, but also localized production in Africa starting in Morocco and Egypt, and potentially in South Africa and Zambia [22]. China’s inactivated vaccines have their advantage to be applied in African countries for safety and easier storage [23]. If the current efforts and commitments can be accelerated, Africa may achieve herd immunity faster than imagined.

## Conclusion

Maintaining commitments to global solidarity and access through initiatives such as COVAX is a viable structure to achieve vaccine equity, resulting in herd immunity in Africa. As long as the virus continues spreading—and mutating—in Africa, the rest of the world is not yet saved. National governments should be accountable for preparing the needed logistical measures and effective cold chain supply structures to ensure an equitable, ethical, and effective vaccine distribution. It is imperative to note that it is high time the African governments owned up responsiblilties concerning the health of her population. Governments should learn from international best practice of prevention and control models and localised these models into the different settings. Even though Africa rely on international aids in time of emergency most of the time, the government should develop financial plans for reserve funds for future emergencies. Initiatives to generate internal revenues should be considered as an option to pay for vaccines in the continent. Community mobilization should be used to discredit false rumour around COVID-19 vaccines and also employed to educate the public on the importance of taking vaccines. Due to the utilization of existing healthcare resources in African countries, stakeholders should integrate the COVID-19 vaccines into the existing healthcare services. This presents a promising strategy to overcome vaccine hesitancy to improve vaccine uptake. African countries should not be left behind in equal vaccine distribution if herd immunity towards COVID-19 is desired globally.

## Data Availability

Not applicable.
